# QTL mapping and GWAS for field kernel water content and kernel dehydration rate before physiological maturity in maize

**DOI:** 10.1038/s41598-020-69890-3

**Published:** 2020-08-04

**Authors:** Shufang Li, Chunxiao Zhang, Ming Lu, Deguang Yang, Yiliang Qian, Yaohai Yue, Zhijun Zhang, Fengxue Jin, Min Wang, Xueyan Liu, Wenguo Liu, Xiaohui Li

**Affiliations:** 1 Crop Germplasm Resources Institute, Jilin Academy of Agricultural Sciences, Kemaoxi Street 303, Gongzhuling, 136100 Jilin Province China; 2 Maize Research Institute, Jilin Academy of Agricultural Sciences, Gongzhuling, 136100 China; 3 College of Agronomy, Northeast Agricultural University, Harbin, 150030 China; 4 Maize Research Center, Anhui Academy of Agricultural Science, Hefei, 230001 China

**Keywords:** Agricultural genetics, Plant breeding, Plant genetics

## Abstract

Kernel water content (KWC) and kernel dehydration rate (KDR) are two main factors affecting maize seed quality and have a decisive influence on the mechanical harvest. It is of great importance to map and mine candidate genes related to KWCs and KDRs before physiological maturity in maize. 120 double-haploid (DH) lines constructed from Si287 with low KWC and JiA512 with high KWC were used as the mapping population. KWCs were measured every 5 days from 10 to 40 days after pollination, and KDRs were calculated. A total of 1702 SNP markers were used to construct a linkage map, with a total length of 1,309.02 cM and an average map distance of 0.77 cM. 10 quantitative trait loci (QTLs) and 27 quantitative trait nucleotides (QTNs) were detected by genome-wide composite interval mapping (GCIM) and multi-locus random-SNP-effect mixed linear model (mrMLM), respectively. One and two QTL hotspot regions were found on Chromosome 3 and 7, respectively. Analysis of the Gene Ontology showed that 2 GO terms of biological processes (BP) were significantly enriched (*P* ≤ 0.05) and 6 candidate genes were obtained. This study provides theoretical support for marker-assisted breeding of mechanical harvest variety in maize.

## Introduction

Maize (*Zea mays* L.) is one of the most important crops in the world. To reduce production costs and increase production efficiency, the whole-process mechanization has become an irreversible trend in world agriculture^1^. Yet mechanical harvest, especially mechanical kernel harvest, remains a bottleneck of whole-process mechanization for maize^2^. The low kernel water content (KWC) at harvest was very important for maize, which could facilitate machinery harvest, shelling efficiency, grain quality and reduce additional drying cost and shrinkage penalties^3-5^. When the KWC at harvest is more than 25%, the breakage rate increases quickly so as to significantly reduce farmers' incomes^6^. Therefore, it is urgent to accelerate the breeding of varieties with low KWC at harvest.

The change in KWC comprises two distinct phases^7,8^. The first phase spans the time from pollination to physiological maturity (PM) and is defined as physiological dehydration. During this phase, kernel water loss is primarily due to dry matter accumulation. The second phase spans the time from PM to harvest and is defined as naturally drying process. In this process, KWC at PM, drying time, and KDR jointly determine KWC at harvest. Previous research has shown that selection based on low ear-moisture content at a specific period after pollination was an effective way to result in low-KWC at harvest^9-11^. The kernel dehydration rate (KDR) is defined as the rate of moisture loss between two adjacent periods after pollination, which is the corresponding index with KWC before PM.

Currently, the genetic mechanism of KWC and KDR is still unclear, making it necessary to further investigate the underlying molecular mechanism and identify relevant major genes. However, prior studies were mostly QTL mapping for KDR after PM and KWC at harvest^3,4,12-17^. Recently, QTL for three traits related with KWCs at 30, 40, 60 and 80 days after pollination (DAP) was conducted by Capelle et al.^3^, using Recombinant Inbred Lines (RILs) F_3:4_ populations derived from a cross between F_2_ and F_252_. Obvious stage-specific QTL were revealed for all traits. QTL for KWCs at 10, 20, 30 and 40 DAP and for KDRs during all periods was conducted by Li et al.^18^, using 258 RILs developed from a cross between N04 and Dan232. The results showed that 45 QTLs were stage/period specific. Besides, there were no other records in the literature regarding QTL for KWC and KDR at different stages before pollination.

In this study, 120 derived double-haploid (DH) lines developed from a cross between two contrasting genotypes, a Tangsipingtou inbred Si287 with low KWC, and a Iodent inbred JiA512 with high KWC were used to map QTLs by genome-wide composite interval mapping (GCIM)^19^ and QTNs by multi-locus random-SNP-effect mixed linear model (mrMLM)^20^, and to mine related candidate genes, which is for KWCs and the corresponding KDRs from 10 to 40 DAP. The results are of important theoretical significance and application value in the mining of candidate gene and the marker-assisted breeding of the field KWC and KDR-related characteristics in maize.

## Results

### Phenotypic evaluation of the DH populations

**Figure 1:**
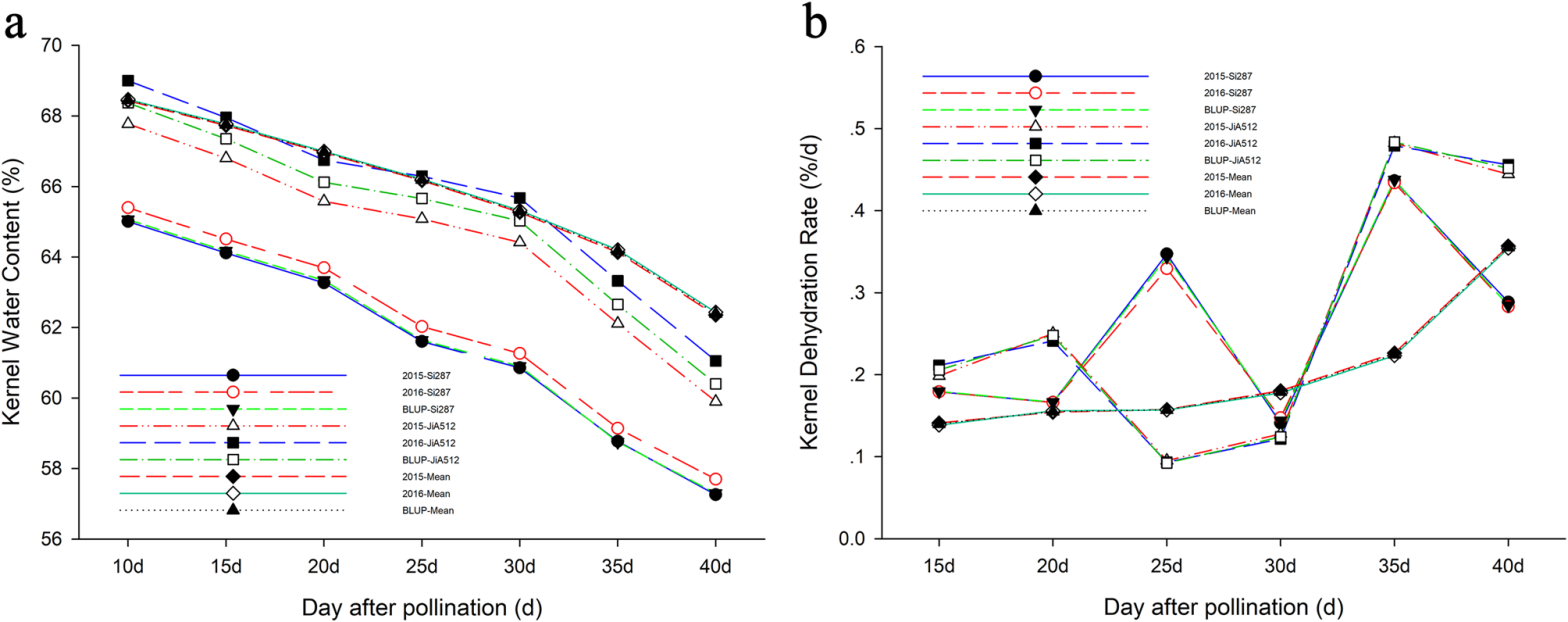
The changing curves for KWCs and KDRs of the parents and the DH population under three environments. (**a**) KWC; (**b**) KDR.

From Table 1 and Supplementary Fig. S1, the KWCs of both parents of the DH line population, Si287 and JiA512, were significantly or extremely significantly different at all the sampling times from 10 to 40 days after pollination in 2015 and 2016. There existed variations in the target traits among different lines, and the coefficients of variation for all the KWCs were less than 10%. The heritability for the KWCs ranged from 77.324 to 79.631%. The correlations between these KWCs for various periods in three environments were more than 90%. From Fig. 1a, we could know that the changing tendency of KWCs in three environments was similar; all continuously declined with increasing days after pollination and generally conformed to linear curve.

**Table 1:** Statistical analysis for KWC and KDR of the parents and the DH population. ^1^SD., standard deviation; ^2^CV., coefficient of variation; ^3^Ske., skewness; ^4^Kur., kurtosis; ^5^Her., heritability; BLUP, best linear unbiased prediction, ** is significant at 0.01 levels.

Traits	ENV	Si287	JiA512	DH population							
				Max	Min	Mean	^1^SD	^2^CV. (%)	^3^Ske	^4^Kur	^5^Her. (%)
KWC10	2015	65.005	67.775**	73.373	63.308	68.435	1.750	2.557	− 0.175	0.239	77.395
	2016	65.400	68.997**	74.096	63.773	68.465	1.887	2.756	− 0.001	− 0.034	
	BLUP	65.064	68.369	73.948	64.285	68.451	1.842	2.691	0.005	− 0.066	
KWC15	2015	64.114	66.803**	72.569	61.933	67.729	1.777	2.623	− 0.244	0.589	77.669
	2016	64.510	67.953**	73.212	63.227	67.773	1.892	2.791	− 0.002	− 0.073	
	BLUP	64.170	67.351	73.061	63.550	67.752	1.856	2.739	− 0.021	0.011	
KWC20	2015	63.268	65.580**	71.766	60.566	66.958	1.830	2.733	− 0.388	0.711	77.667
	2016	63.694	66.748**	72.452	62.634	66.993	1.878	2.803	− 0.036	− 0.135	
	BLUP	63.339	66.123	72.309	62.194	66.977	1.876	2.802	− 0.112	− 0.002	
KWC25	2015	61.602	65.082**	70.872	59.652	66.170	1.830	2.766	− 0.323	0.863	77.324
	2016	62.025	66.292**	71.555	61.932	66.209	1.872	2.827	− 0.060	− 0.194	
	BLUP	61.637	65.657	71.410	61.256	66.191	1.870	2.826	− 0.101	0.010	
KWC30	2015	60.855	64.418**	70.274	58.531	65.270	1.898	2.908	− 0.335	0.829	78.664
	2016	61.261	65.675**	70.902	60.573	65.320	1.961	3.001	− 0.070	− 0.202	
	BLUP	60.897	65.027	70.784	60.148	65.296	1.950	2.986	− 0.118	0.035	
KWC35	2015	58.773	62.112**	68.850	57.721	64.136	1.860	2.900	− 0.254	0.564	78.023
	2016	59.140	63.323**	69.286	59.545	64.204	1.936	3.016	− 0.078	− 0.222	
	BLUP	58.752	62.652	69.259	59.373	64.172	1.918	2.989	− 0.080	− 0.092	
KWC40	2015	57.257	59.899**	67.706	55.222	62.352	1.990	3.191	− 0.382	0.716	79.631
	2016	57.696	61.051**	68.137	56.787	62.434	2.092	3.352	− 0.171	− 0.190	
	BLUP	57.298	60.399	68.120	56.818	62.395	2.062	3.305	− 0.198	0.005	
KDR15	2015	0.179	0.199**	0.296	0.036	0.141	0.058	40.928	0.392	0.074	71.773
	2016	0.179	0.211**	0.302	0.030	0.138	0.057	40.894	0.368	0.184	
	BLUP	0.179	0.206	0.298	0.032	0.140	0.057	41.007	0.365	0.151	
KDR20	2015	0.166	0.250**	0.315	0.037	0.154	0.069	44.562	0.532	− 0.516	66.746
	2016	0.166	0.241**	0.310	0.035	0.156	0.067	43.025	0.502	− 0.592	
	BLUP	0.166	0.248**	0.316	0.036	0.155	0.069	44.308	0.511	− 0.586	
KDR25	2015	0.347	0.095**	0.360	0.044	0.157	0.075	47.630	0.763	− 0.194	63.235
	2016	0.329	0.093**	0.341	0.051	0.157	0.071	45.478	0.798	− 0.300	
	BLUP	0.344	0.092	0.357	0.044	0.157	0.074	47.135	0.764	− 0.278	
KDR30	2015	0.141	0.128*	0.435	0.030	0.180	0.070	39.036	0.748	1.243	72.711
	2016	0.147	0.122*	0.427	0.034	0.178	0.071	39.919	0.462	1.408	
	BLUP	0.144	0.124	0.433	0.029	0.179	0.071	39.567	0.659	1.227	
KDR35	2015	0.437	0.482**	0.482	0.023	0.226	0.095	42.057	0.278	− 0.224	73.295
	2016	0.434	0.479**	0.460	0.045	0.223	0.090	40.330	0.351	− 0.214	
	BLUP	0.438	0.484	0.464	0.042	0.225	0.093	41.388	0.314	− 0.240	
KDR40	2015	0.288	0.444**	0.581	0.133	0.357	0.104	29.182	− 0.175	− 0.800	71.227
	2016	0.283	0.456**	0.591	0.142	0.354	0.102	28.768	− 0.099	− 0.763	
	BLUP	0.284	0.452	0.590	0.134	0.356	0.104	29.285	− 0.149	− 0.782

From Table 1 and Supplementary Fig. S2, the KDRs of Si287 and JiA512, were significantly or extremely significantly different at a majority of the above sampling times in 2015 and 2016. There existed variations in the target traits among different lines, and the coefficients of variation for all the KDRs were more than 10% and ranged from 28.76 to 47.63%. The heritability for the KDRs ranged from 63.235 to 73.295%. In the DH population, only the kurtosis for KWC30 was > 1, and the absolute values of skewness and kurtosis for other traits were < 1, which met the QTL mapping requirements for mapping studies. The KDRs for various periods were different among different lines of the DH population, but the correlations between these indicators were poor. From Fig. 1b, we could also know that the changing tendency of KDRs in the mean environment was continuously increased with increasing days after pollination.

### Genetic map construction

Using the Axiom Maize55K^21^ chip and upon filtration per the criteria described in section “DNA extraction and genotype analysis”, there remained 12,861 polymorphic SNPs. The bin function in IciMapping software was used to delete redundant markers, the recombination frequency between them will be estimated as 0. A genetic linkage map containing 1702 markers was eventually obtained. 1702 markers covered 1,309.02 cM on 10 chromosomes (Chr.) with an average marker interval of 0.77 cM (Supplementary Fig. S3). Total length of the map for each chr. ranged from 98.96 cM (chr.8) to 234.93 cM (chr.1). Chr.4 and 1 had the least (115 markers) and most (335 markers) markers, respectively; chr.3 and 4 had the minimum (0.63 cM) and maximum (0.97 cM) average marker-intervals, respectively. Only one gap ≥ 10 cM existed on Chr.4 (10.46 cM); 1,680 gaps ≤ 5 cM existed (Table 2).

**Table 2:** Characteristics of the high-density genetic map derived from a cross between Si287 and JiA512.

Chr. no.	Number of markers	Physical distance (Mb)	Genetic distance (cM)	Avg distance between markers (cM)	Gap (cM)		
					≤ 5	≥ 10	Max
1	335	301.28	234.93	0.70	333	0	6.41
2	228	236.71	181.20	0.80	226	0	8.04
3	165	232.16	103.39	0.63	164	0	3.41
4	115	239.69	107.86	0.97	113	1	10.46
5	183	217.76	121.19	0.67	181	0	5.94
6	153	169.05	129.98	0.86	152	0	4.41
7	168	173.19	123.02	0.74	165	0	5.99
8	118	175.34	98.96	0.85	116	0	7.13
9	118	156.87	100.20	0.86	115	0	6.20
10	119	149.46	108.47	0.92	115	0	5.99
Total	1702	2051.52	1,309.02	0.77	1,680	1	10.46

### QTL mapping for KWCs and KDRs

**Figure 2:**
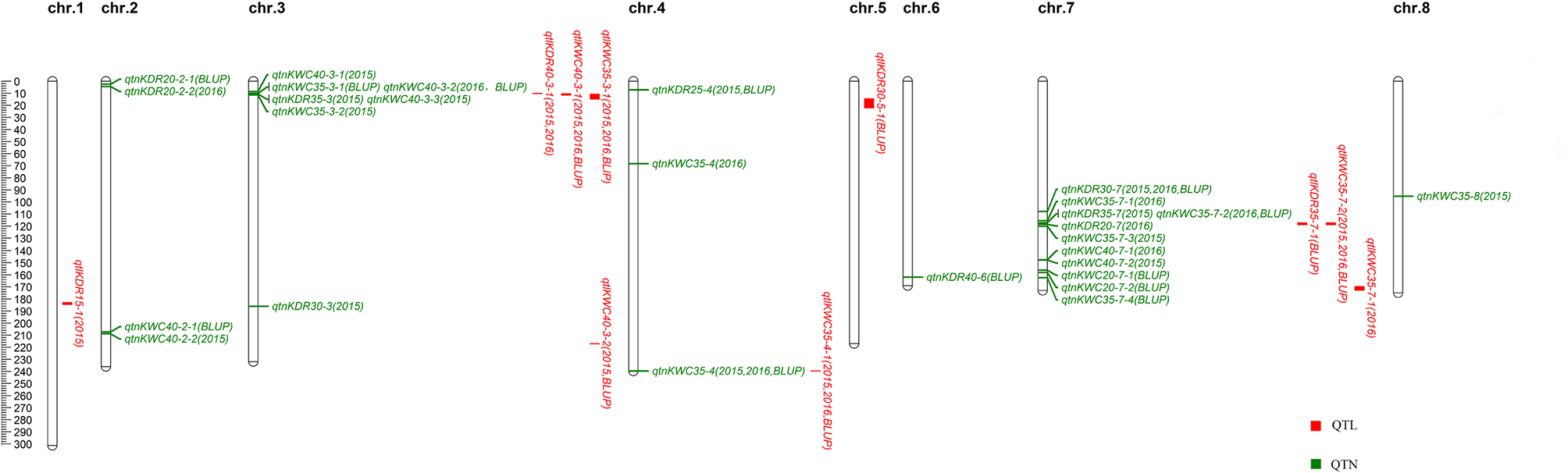
Chromosomes location of QTLs and QTNs for KWCs and KDRs in three environments. Red is for the QTLs, green is for the QTNs.

The GCIM model detected 10 additive QTLs related to KWC and KDR (Table 3, Fig. 2, Supplementary Figs. S4-S6), in which 575 candidate genes were annotated (Supplementary Table S1) and 6 QTLs were detected in two or three environments. These QTLs were distributed on Chr. 1, 3–5, 7 and had an LOD range of 2.54–3.87 and could explain 3.06–16.03% of the phenotypic variation (PVE). For 6 QTLs related to KWC, *qKWC35-3-1*, *qtlKWC35-7-1* and *qtlKWC40-3-2* derived from the maternal line Si287, which had an LOD range of 2.76–3.41 and the range of PVE was 3.72–8.85%; *qtlKWC35-4-1*, *qtlKWC35-7-2* and *qtlKWC40-3-1* derived from the paternal line JL001, which had an LOD range of 2.76–3.41 and the range of PVE was 3.06–12.10%. For 4 QTLs related to KDR, *qtlKDR30-5-1*, *qtlKDR35-7-1* and *qtlKDR40-3-1* derived from the maternal line Si287, which had an LOD range of 2.54–3.47 and the range of PVE was 7.24–12.80%; *qtlKDR15-1* derived from the paternal line JL001, which had an LOD 2.64 and the PVE was 16.03%. *qtlKWC35-7-2* and *qtlKDR35-7-1* were located at the same interval; *qtlKWC35-3-1*, *qtlKWC40-3-1* and *qtlKDR40-3-1* had an interval adjacent to each other. The above results are consistent with previous studies, which indicated that KDR is a maternal effect^24^ but has a paternal effect as well^25^.

**Table 3:** QTL mapping for KWCs and KDRs in DH population. 11: 2015; 2: 2016; 3: BLUP.

QTL	Trait	Chr	Pos. (cM)	Add	LOD	PVE (%)	Bin marker interval	Confidence interval (Mb)	^1^ENV	Previous QTLs
qtlKWC35-3-1	KWC35	3	3.19	0.64–0.76	2.76–3.41	4.0–8.85	AX-90796489–AX-91556213	10.69–14.85	1, 3	qKdr-3-1 Wang et al.^22^
qtlKWC35-4-1	KWC35	4	107.86	− 0.64 to − 0.90	2.88–3.70	4.15–12.10	AX-86314360–AX-91641504	239.54–239.69	1, 2, 3	
qtlKWC35-7-1	KWC35	7	46.56	0.72	3.22	5.20	AX-91743846–AX-91744474	169.62–172.87	3	
qtlKWC35-7-2	KWC35	7	104.91	-0.63	2.67	3.98	AX-91734685–AX-91411127	117.27–118.92	3	Rate_30_40_2 Capelle et al.^3^
qtlKWC40-3-1	KWC40	3	2.04	− 1.75 to − 1.91	2.61–3.87	3.06–9.66	AX-86268070–AX-86262944	10.39–11.66	1, 2, 3	
qtlKWC40-3-2	KWC40	3	85.67	1.93	2.87	3.72	AX-90851052–AX-90851146	216.93–217.32	2	qKdr-3-6 Wang et al.^22^
qtlKDR15-1	KDR15	1	150.95	− 0.40	2.54	16.03	AX-91441400–AX-86240805	182.88–184.65	1	
qtlKDR30-5-1	KDR30	5	91.31	0.04–0.05	2.59–2.71	7.24–11.76	AX-91646000–AX-90609735	14.49–22.21	1, 2	
qtlKDR35-7-1	KDR35	7	104.91	0.10–0.11	3.30–3.47	12.60–12.80	AX-91734685–AX-91411127	117.27–118.92	1, 2, 3	
qtlKDR40-3–1	KDR40	3	0.58	0.29–0.32	2.54–2.73	9.80–9.98	AX-90796530–AX-86268070	10.02–10.39	1, 2, 3	qFkdr3a Qian et al.^23^

### GWAS for KWCs and KDRs

Structure 2.3.4 software^26^ was used to calculate the population structure (Q value),by setting the range of K value to 1–10 and based on the kinship and ΔK value of the parents Si287 and JiA512, K = 2 was specified (Supplementary Fig. S7). Using population structure (Q) and kinship (K) as covariates, the Q + K model in the mixed linear model mrMLM was used to perform GWAS for KWC and KDR. A total of 27 QTNs associated with KWC and KDR were detected (Table 4, Fig. 2, Supplementary Fig. S8) and were distributed on Chr. 2–4 and Chr.6–8, and the range of PVE was 0.34–11.58%, in which 7 QTNs were detected by more than two environments or two methods. *qtnKDR25-4*, *qtnKWC35-7-2* and *qtnKWC40-3-2* were detected by two environments and the range of PVE was 2.88–8.23%. *qtnKWC35-8* and *qtnKWC40-3-3* were respectively detected by two or three models and the range of PVE was 4.56–11.58%. *qtnKDR30-7* and *qtnKWC35-4* were respectively detected by two or three models in three environments.

**Table 4:** QTNs for KWCs and KDRs based on six models. ^1^1: 2015; 2: 2016; 3: BLUP. ^2^1: pLARmEB; 2: ISIS EM-BLASSO; 3: mrMLM; 4: FastmrMLM; 5: FASTmrEMMA; 6: pKWmEB.

QTN	Trait	SNP	Chr	Pos. (Mb)	QTN effect	LOD score	PVE (%)	^1^ENV	^2^Method	Previous QTLs
qtnKWC15-4	KWC15	AX-90873933	4	68.28	− 0.34	3.33	5.17	2	1	
qtnKWC20-7-1	KWC20	AX-91062728	7	156.22	− 0.31	3.46	7.53	3	2	
qtnKWC20-7-2	KWC20	AX-91741712	7	158.15	− 0.31	4.11	7.27	3	2	
qtnKWC35-3-1	KWC35	AX-86281780	3	10.10	0.50	3.59	5.73	3	2	*qFkdr3a*, *qFkdr3c* Qian et al.^23^
qtnKWC35-3-2	KWC35	AX-86310397	3	11.54	− 0.70	4.58	7.55	1	2	*qFkdr3a*, *qFkdr3c* Qian et al.^23^
qtnKWC35-4	KWC35	AX-91641504	4	239.69	− 0.38 to − 0.64	3.02–3.82	3.22–5.93	1, 2, 3	1, 2, 4	
qtnKWC35-7-1	KWC35	AX-86318553	7	115.58	− 0.64	3.18	5.66	2	2	
qtnKWC35-7-2	KWC35	AX-86251963	7	117.34	− 0.52 to − 0.76	3.66–4.12	5.88–7.94	2, 3	1	
qtnKWC35-7-3	KWC35	AX-91357015	7	120.29	− 0.66	3.75	6.52	1	1	
qtnKWC35-7-4	KWC35	AX-91064514	7	162.43	− 0.46	3.04	4.82	3	2	
qtnKWC35-8	KWC35	AX-86253350	8	95.29	0.54	3.47–3.53	4.56–4.79	1	1, 2	*Water_80_5* Capelle et al.^3^
qtnKWC40-2-1	KWC40	AX-123946682	2	207.29	1.61	3.06	6.34	3	1	*qKdr-2-2* Wang et al.^22^
qtnKWC40-2-2	KWC40	AX-90785588	2	208.86	1.95	3.75	7.75	1	2	*qKdr-2-2* Wang et al.^22^; *q9GDR13-2-1* Li et al.^18^
qtnKWC40-3-1	KWC40	AX-86288465	3	8.60	− 1.53	3.05	6.31	1	1	
qtnKWC40-3-2	KWC40	AX-86281780	3	10.10	1.61	3.24–3.31	8.11–8.23	2, 3	2	*qFkdr3a*, *qFkdr3c* Qian et al.^22^
qtnKWC40-3-3	KWC40	AX-91555465	3	10.57	− 1.64 to − 2.13	3.04–3.77	6.88–11.58	1	2, 3, 4	*qFkdr3a*, *qFkdr3c* Qian et al.^22^
qtnKWC40-7-1	KWC40	AX-91060390	7	147.62	− 1.36	3.03	4.77	2	2	
qtnKWC40-7-2	KWC40	AX-91739850	7	147.82	− 1.77	4.40	8.09	1	2	
qtnKDR20-2-1	KDR20	AX-86283442	2	2.54	− 0.01	3.88	0.99	3	2	
qtnKDR20-2-2	KDR20	AX-90731189	2	4.50	− 0.01	4.42	0.34	2	1	
qtnKDR20-7	KDR20	AX-90636690	7	118.62	0.03	3.63	4.04	2	2	
qtnKDR25-4	KDR25	AX-86312325	4	7.18	0.02–0.03	3.03–7.73	2.88–3.00	1, 2	1	
qtnKDR30-3	KDR30	AX-90842115	3	186.13	0.02	13.93	1.89	1	1	
qtnKDR30-7	KDR30	AX-116872292	7	107.87	0.01–0.02	3.48–11.09	0.46–3.63	1, 2, 3	1, 2	
qtnKDR35-3	KDR35	AX-91555465	3	10.57	0.05	4.39	2.71	1	1	*qFkdr3a*, *qFkdr3c* Qian et al.^22^
qtnKDR35-7	KDR35	AX-86251963	7	117.34	0.09	4.68	9.37	1	2	
qtnKDR40-6	KDR40	AX-91450874	6	161.99	− 0.23	3.41	5.92	3	2	*qKdr6-1* Zhang^27^

### Co-mapping analysis of QTLs and significantly associated loci in GWAS and candidate gene mining

The 5 KWC-related QTNs detected by the mrMLM model in the GWAS were consistent with the intervals of KWC- and KDR-related QTLs (Fig. 2). *qtnKWC35-3-1* and *qtnKWC40-3-2* were within the physical interval of *qtlKDR40-3-1*. *qtnKDR35-3* and *qtnKWC40-3-3* were within the physical interval of *qtlKWC40-3-1*. *qtnKWC35-3-2* was within the physical interval of *qtlKWC35-3-1*. *qtnKWC35-4* was within the physical interval of *qtlKWC35-4-11*. *qtnKDR35-7*, *qtnKWC35-7-2* and *qtnKDR20-7* were within the physical interval of *qtlKDR35-7-1* and *qtlKWC35-7-2*. After mapping the 10 QTLs and 27 QTNs on the B73 genetic map, there were 3 maize KWC- and KDR-related hotspot regions on Chr.3 and 7, corresponding to 8,596,700–11,655,573 bp, 117,271,199–118,924,581 bp and 162,434,519–172,868,044 bp.

**Figure 3:**
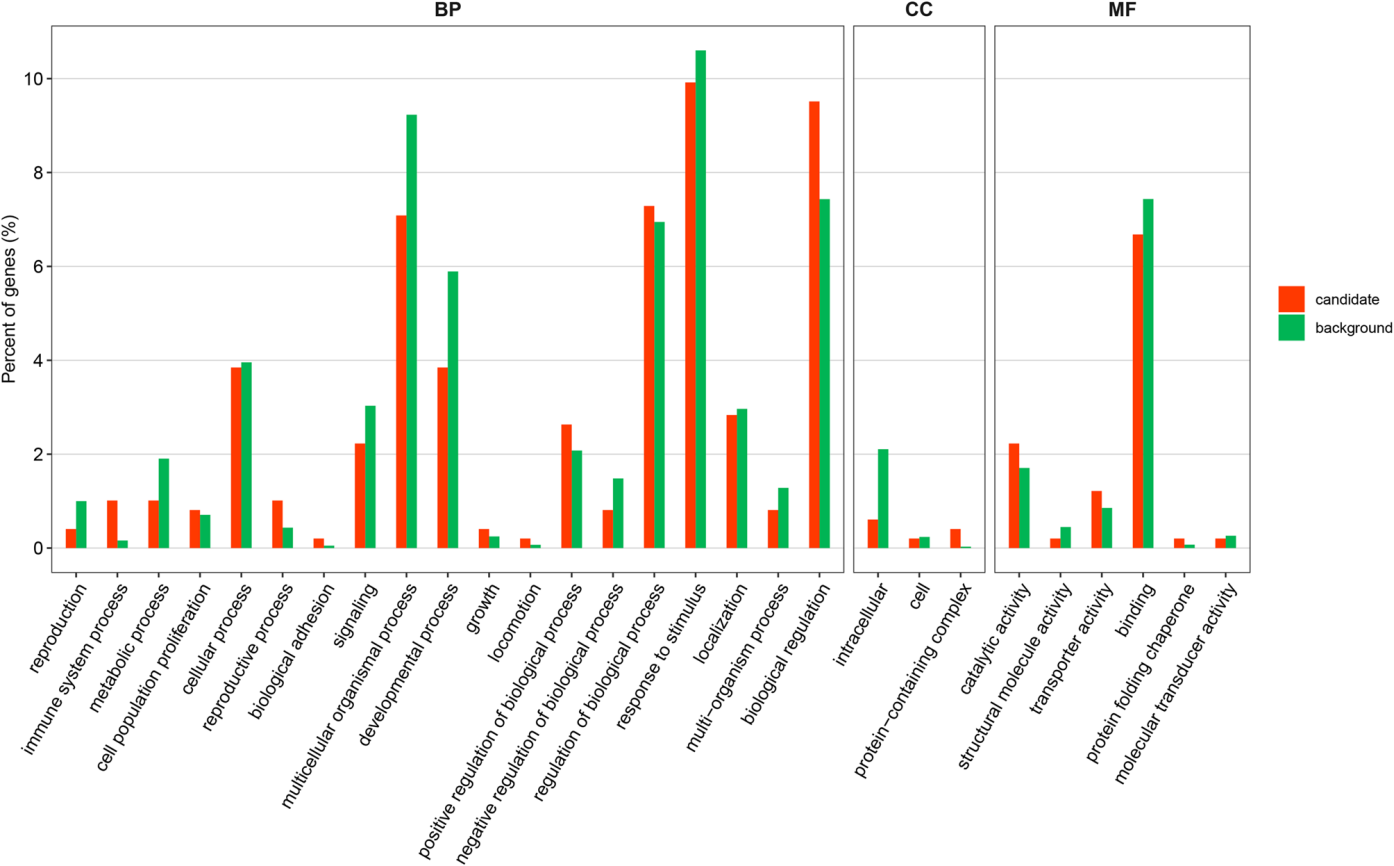
The annotation of the common candidate genes in GO analysis.

To explore genes potentially related to KWCs and KDRs in maize, we analyzed the above 3 hotspot regions. The 3 regions have 98, 33, and 363 genes, respectively. These genes are annotated for enrichment analysis using AGRIGO V2 software (https://systemsbiology.cau.edu.cn/agriGOv2/index.php). According to the functions in the GO database, the terms can be grouped into 3 categories (Fig. 3): molecular function (MF), cellular component (CC) and biological process (BP). For MF, there are 6 terms; For CC, there are 3 terms and for BP, there are 19 terms. 2 GO terms of BP were significantly enriched (*P* ≤ 0.05) (Supplementary Table S2), the KWCs and KDRs may be related to certain BP terms. 6 candidate genes were obtained (Supplementary Table S3).

## Discussion

The availability of a reliable methodology to measure KWC under field conditions is a bottleneck in selection for KDR^28^. The traditional oven method is destructive and not suitable for rapid detection of KWC. Instead, a moisture determination metric, which reveals kernel moisture via detection of electric capacity variation, has been developed^29^. This method was listed in the International Seed Testing Protocol in 2003. The hand-held moisture meter has been reported to be useful for selecting and evaluating genetic materials^4,11,12,23,28,30^. In this study, an SK-300 probe (manufactured by Harbin Yuda Electronic Technology Co., Ltd., China) was used to measure KWC.

Many studies suggested that there is close relation between KWC and KDR and environmental factors including air temperature, air humidity, rainfall, etc^30-32^. Hence, the following measures were taken in this study so as to avoid the effect of the environmental factors on KWC and ensure the determination accuracy. (1) To avoid border effects, for each plot, 2 border rows and the first 2 plants at each end of the middle 3 rows were not used for future determination. (2) The ears were bagged before silking and pollinated by hand. One week later, the bags were removed and 5 tested ears were randomly selected, tagged and labeled in each plot. (3) The measurement time was established for 9:00 A.M. to remove the effect of the dew and the difference of measurement time. (4) If it rains, the KWC was measured after wiping the outer bracts of the ears to eliminate the effect of the rainfall.

RIL and DH population are all permanent mapping populations. The former has a high degree of recombination, but the constructed period is very long and dominant effect couldn`t be estimated. The latter has a bad degree of recombination, but the plants are homozygous and could be used to study the interactions between genotypes and environments.

The quality of the genetic map directly affects the accuracy of QTL mapping. Increasing marker density can improve the resolution of genetic map^33-35^. With the development of high-throughput sequencing and re-sequencing of the whole genome of the B73, numerous SNP markers have become effective means for constructing high-density genetic maps for maize^36,37^. SNPs provide abundant genetic variation loci at the genome level, which greatly improves genome coverage and marker saturation^38-40^. In previous studies, very significantly distorted markers were discarded in the construction of linkage maps, but the more markers increase total genetic distance and marker density on the chromosome^41^, and use of fewer distorted markers in all the RILs decreases the impact of distorted marker on map construction^42,43^. In this study, the Axiom Maize55K chip was used for genotyping DH lines and their parents to screen out 12,861 polymorphic markers, and upon removing redundant markers that the recombination frequency between them will be estimated as 0, a linkage map containing 1,702 markers including segregation distortion markers was obtained, with a total full length of 1,309.02 cM and an average map distance of 0.77 cM.

Linkage analysis was the most widely-used method in QTL mapping, which included the composite interval mapping (CIM), the inclusive composite interval mapping (ICIM), etc. Up to now, most of the numerous QTLs have small effects on complex traits^44^, and some are closely linked^45^. Although QTL mapping has proven to be useful for detecting major QTL with relatively large effects, it may lack power in accurately modeling small-effect QTL^46^. To address this issue, Genome-wide association study (GWAS) was developed to reconsider the model and improve the way that polygenic background is controlled. The GWAS data often includes a large number of markers, making co-factor selection infeasible. Thus, polygenic effects are often fitted to a mixed linear model to capture the genomic background information^47,48^. This treatment can help us improve the methods of QTL mapping, and overcome the subjectivity nature of the CIM in co-factorselection^49,50^. A series of simulated and real datasets was used to compare the different methods. The results showed that GWAS analysis had higher power in QTL detection, greater accuracy in QTL effect estimation, and stronger robustness under various backgrounds as compared with the CIM and empirical Bayes methods^19^.

KWC and KDR after field pollination in maize are complex quantitative traits susceptible to environmental conditions and are controlled by multiple genes^8,51^. As maize KWC and KDR are affected by additive genetic effects and have high heritability^7,51-55^, it is feasible to carry out mapping of major QTLs for KWC and KDR, to mine candidate genes and to develop practical functional markers for marker-assisted selection. In this study, 10 QTLs and 27 QTNs were detected, in which 4 QTLs and 29 QTNs were consistent with previous studies. The others in this study have not been reported. 2 GO terms of BP were significantly enriched (P ≤ 0.05), the KWCs and KDRs may be related to certain BP terms. 6 candidate genes were obtained, in which Zm00001d022326 coded gibberellin receptor GID1L2 (*Zea mays*). These co-located QTL are reliable and will be valuable for marker assisted selection in maize genetic improvement.

## Conclusions

The KWCs and the KDRs of both parents of the DH line population, Si287 and JiA512, were significantly or extremely significantly different at the sampling times from 10 to 40 days after pollination in 2015 and 2016. There existed variations in the target traits among different lines. The heritability for the KWCs and KDRs was very high. 10 quantitative trait loci (QTLs) and 27 quantitative trait nucleotides (QTNs) were detected by genome-wide composite interval mapping (GCIM) and multi-locus random-SNP-effect mixed linear model (mrMLM), respectively. One and two QTL hotspot regions were found on Chromosome 3 and 7, respectively. Analysis of the Gene Ontology showed that 2 GO terms of biological processes (BP) were significantly enriched (*P* ≤ 0.05) and 6 candidate genes were obtained. This study provides theoretical support for marker-assisted breeding of mechanical harvest variety in maize.

## Materials and methods

### Plant materials

Si287 (low KWC) and the self-selection line JiA512 (high KWC) were selected as parents based on their similitude in time to flowering and their difference in KDR, which were part of the Tangsipingtou and Iodent heterotic groups, respectively. Specifically, Si287 was the maternal of the maize hybrid Jidan 27, which has been continuously grown for fifteen years in Heilongjiang Province, China with the most annual planting acreage, reaching up to 160,000 hectares. A DH population of 120 lines was developed from a cross between Si287 (maternal) and JiA512 (paternal).

The development of DH population was briefed as follows: In the summer of 2013, at the Gongzhuling (Jilin Province, China) Experimental Base of the Jilin Academy of Agricultural Sciences (JAAS), Si287 and JiA512 were crossed to obtain F1. In the winter of 2013, at the Ledong (Hainan Province, China) winter nursery of JAAS, the F1 plants as the maternal parent were made to obtain induced progenies using the induction line “Jiyou 101” as the paternal parent. In the summer of 2014, at the Gongzhuling Experimental Base, the induced progenies were chromosome-doubled using colchicine, followed by kernel identification; upon field identification and selection, 120 DH lines were obtained. In the winter of 2014, at the Ledong winter nursery, the 120 DH lines were multiplied in large number and used in subsequent experiments.

### Field design and phenotypic measurements

The 120 DH lines and their parents were sown on April 25, 2015 and on April 29, 2016 at Gongzhuling (124°47′ N and 43°27′ E), with a final plant density of 75,000 plants ha^-1^. A randomized block design with three replications was adopted in two environmental evaluations. In both years, each plot had 5 rows, with a row length of 5 m, row spacing of 0.65 m, plant spacing of 0.20 m and plot area of 16.25 m^2^. The field management in both years was the same. To avoid border effects, for each plot, 2 border rows and the first 2 plants at each end of the middle 3 rows were not used for future trait determination.

The ears were bagged before silking (50% of plants in the row having extruded silks). Then the bagged ears were pollinated by hand (Supplementary Table S4). One week later, the bags were removed and 5 tested ears were randomly selected, tagged and labeled in each plot. The water content was recorded from 10 to 40 day after pollination, with one measurement of every 5 days. At 9:00 a.m., per the method published by Reid et al.^29^, for each ear, a SK-300 probe for water content measurement (manufactured by Harbin Yuda Electronic Technology Co., Ltd., China) was used to pierce into kernels after penetrating the bract leaves in the middle of the ear.

KWCs on day 10, 15, 20, 25, 30, 35, 40 after pollination were measured, which were designated as KWC15, KWC20, KWC25, KWC30, KWC35 and KWC40, respectively. KDRs were then calculated based on KWCs for 2 adjacent times. KDR = (KWC at a given time—KWC at the next time)/number of days during the time span. The KDRs for the 6 time spans (namely, 10–15, 15–20, 20–25, 25–30, 30–35 and 35–40 days after pollination) were respectively denoted as KDR15, KDR20, KDR25, KDR30, KDR35, and KDR40. ${\text{CV}}\left( {{\text{Coefficient}}\;{\text{of}}\;{\text{variation}}} \right) = {\text{SD}}\left( {{\text{Standard}}\;{\text{Deviation}}} \right)/{\text{Mean}}$. One-way analysis of variance (ANOVA) between parents and the descriptive statistics for the DH population was conducted using SPSS 22.0 (SPSS, Chicago, IL, United States). R software was used to analyze the correlation between various traits. The best linear unbiased predictions (BLUPs) for each trait of 2 years were calculated using the R package Lme4^56^ with the following model: ${\text{y}} = {\text{Imer}}\left( {{\text{Trait}}\sim \left( {1|{\text{Genetype}}} \right) + \left( {1|{\text{Year}}} \right)} \right)$.

### DNA extraction and genotype analysis.

Genomic DNA of 120 DH lines and their parents were extracted from young leaves by a modified cetyltrimethyl ammonium bromide (CTAB) method. DNA quality was determined by agarose gel electrophoresis (0.8%) and spectrophotometry (NanoDrop 2000). Genotyping was performed using an Axiom Maize55K biochip^21^ from CapitalBio Corporation (Beijing, China).

The Axiom Maize55K biochip contains 55,229 single-nucleotide polymorphisms (SNPs). Based on the Affy Axiom Array 2.0 platform, the 120 DH lines and their parents were genotyped. Upon genotyping, the original data were filtered based on the following criteria: (1) minor allele frequency (MAF) > 0.05 and missing genotype rate < 0.1 (24,622 remained); (2) missing SNP loci for any or both of the parents (24,481 remained); (3) no polymorphisms at loci between parents (17,124 remained); and 4) heterozygous loci for any of the parents (12,861 remained). The PLINK program (version 1.9)^57^ was obtained SNPs with MAF > 0.05 and missing genotype rate < 0.1 for association analyses.

### Genetic map construction

The bin function in QTL IciMapping V4.1^58^ software was used to delete redundant SNP markers, the recombination frequency between them will be estimated as 0, and the remaining markers were bin markers and used to construct a genetic linkage map. The threshold value of the logarithm of odds (LOD) was set as 3.0, and the Kosambi function^59^ was used to start the program. Centi-Morgan (cM) was used to represent the intervals of markers on the map.

### QTL mapping and comparison with previous studies

All the above phenotypes, along with marker genotypic information and linkage maps, were used to identify QTLs using genome-wide composite interval mapping (GCIM)^19^, implemented by the software program QTL.gCIMapping.GUI^60^, where the threshold for significant QTL was set at LOD = 2.5 and the walking speed was 1 cM. Considering that all potential QTLs were selected in the first stage, we decided to place a slightly more stringent criterion of 0.000691, which is converted from LOD score 2.50 of the test statistics using ${\text{P}}_{{\text{r}}} \left( {\upchi _{{\text{v}}}^{2} > 2.50 \times 4.605} \right) = 0.000691$. The above-mentioned QTL nomenclature refers to the method in McCouch et al.^61^. The QTL nomenclature was designated as: qtl + trait abbreviation + chromosome number + QTL number. MapChart 2.3 software^62^ was used to draw genetic linkage maps and label QTLs. When a QTL in the current study shared the same physical region as the previous QTL, it was regarded as a repeated identification of the previous QTL; otherwise, the current QTL was regarded as a new one.

### Genome-wide association studies

All the above phenotypic and genotypic information in the above mapping population was used to detect QTNs using the mrMLM^20^, FASTmrEMMA^63^, FASTmrMLM^64^, pLARmEB^65^, pKWmEB^66^ and ISIS EM-BLASSO^67^ approaches, implemented by the software program mrMLM v4.0. The aboved six methods belonged to the “mrMLM” software package, which was developed by Professor Yuanming Zhang from College of Plant Science and Technology of Huazhong Agricutural University. The unified parameter settings for the six methods were as follows: (1) the Q + K model was used, in which the population structure value Q was calculated by Structure 2.3.4 software23 and the kinship value K was analysed by the “mrMLM” software package; and (2) the significant threshold FPR (the false positive rate) value was set as 0.0002 (LOD = 3.0), which was calculated as the ratio of the number of false positive effects to the total number of zero effects considered in the full model. In addition, while using mrMLM and FASTmrEMMA, the search radius of candidate genes was specified as 20 kb; using pLARmEB, 50 potential association loci were selected on each chromosome. The QTN nomenclature was designated as: qtn + trait abbreviation + chromosome number + QTN number.

### Candidate genes identification

All QTLs and QTNs related to maize KWC and KDR detected by QTL gCIMapping software and the "mrMLM" software package were mapped to the maize reference genome B73 RefGen_V4, and candidate genes were identified in hotspot regions where QTL intervals overlapped QTNs. The resultant candidate genes were subjected to Gene Ontology (GO) enrichment analysis for selecting candidate genes related to maize KWC and KDR.

## Supplementary information

Supplementary Figure S1

Supplementary Figure S2

Supplementary Figure S3

Supplementary Figure S4

SSupplementary Figure S5

Supplementary Figure S6

Supplementary Figure S7

Supplementary Figure S9

Supplementary Table S1

Supplementary Table S2

Supplementary Table S3

Supplementary Table S4
