# Supplementary figures and images for: QTL mapping and GWAS for field kernel water content and kernel dehydration rate before physiological maturity in maize
Source: Sci Rep. 2020 Aug 4;10:13114. doi: 10.1038/s41598-020-69890-3 (PMC7403598; doi:10.1038/s41598-020-69890-3)

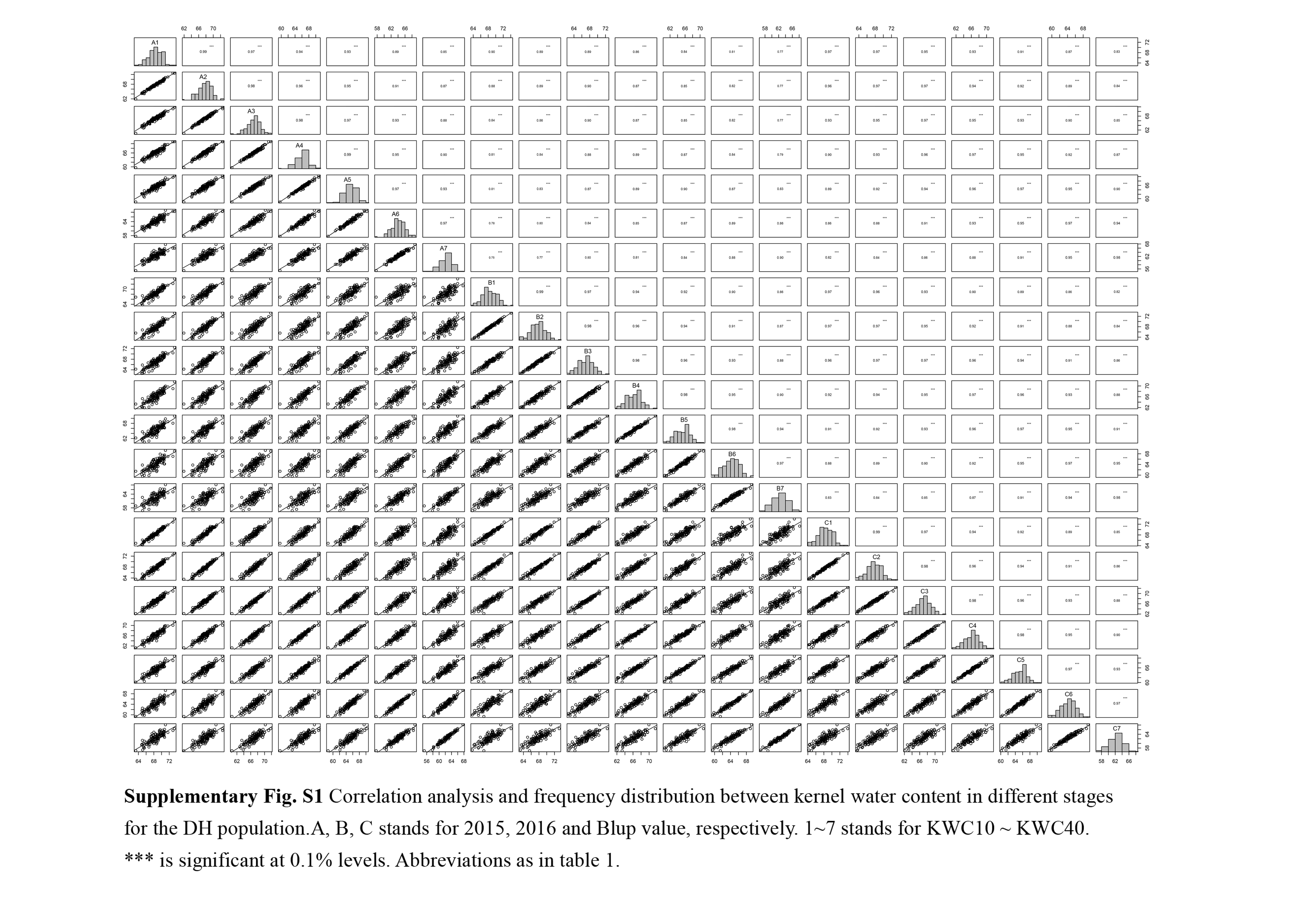

Supplement: Supplementary file 1 — Supplementary Figure S1 [file 41598_2020_69890_MOESM1_ESM.tif]

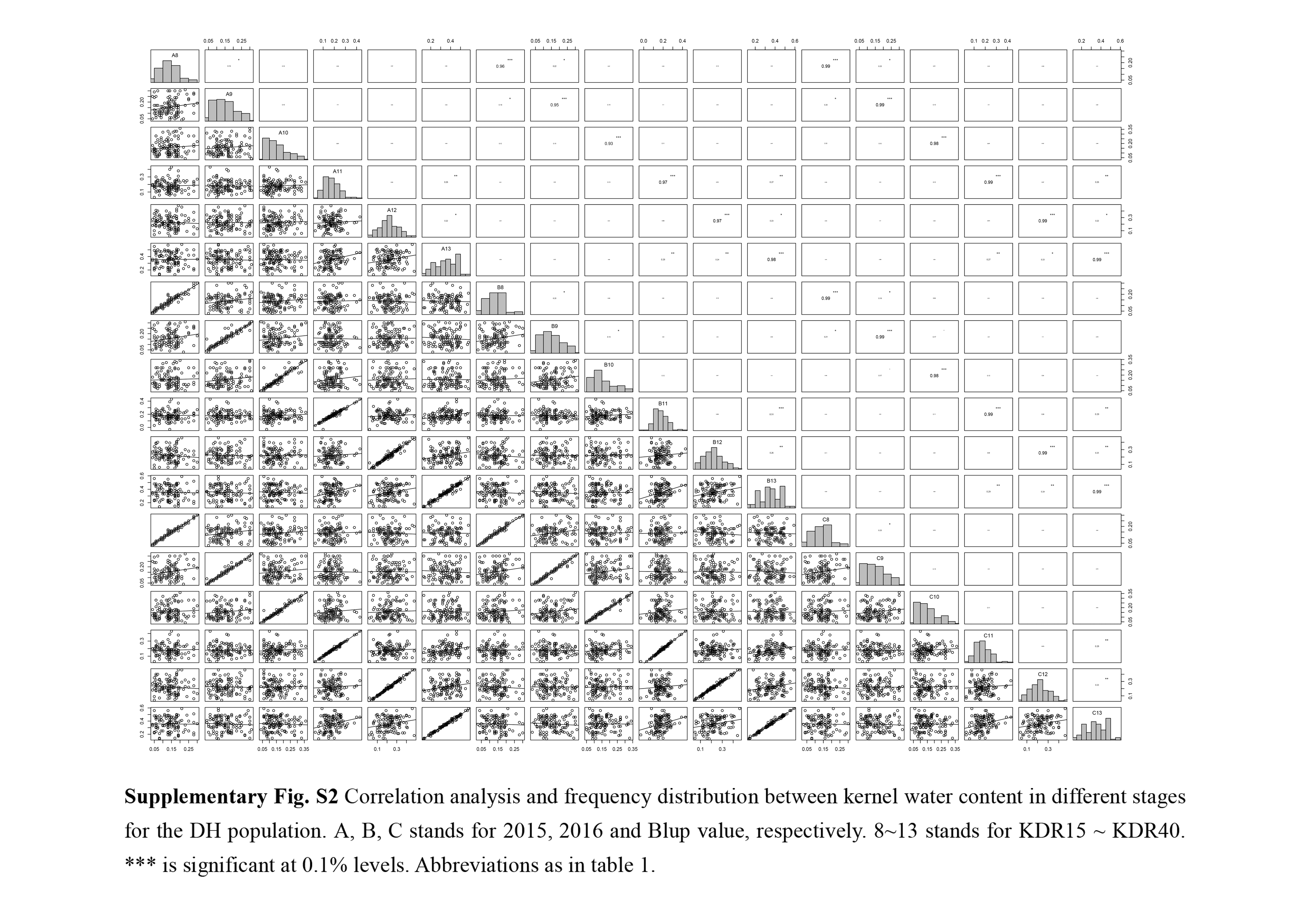

Supplement: Supplementary file 2 — Supplementary Figure S2 [file 41598_2020_69890_MOESM2_ESM.tif]

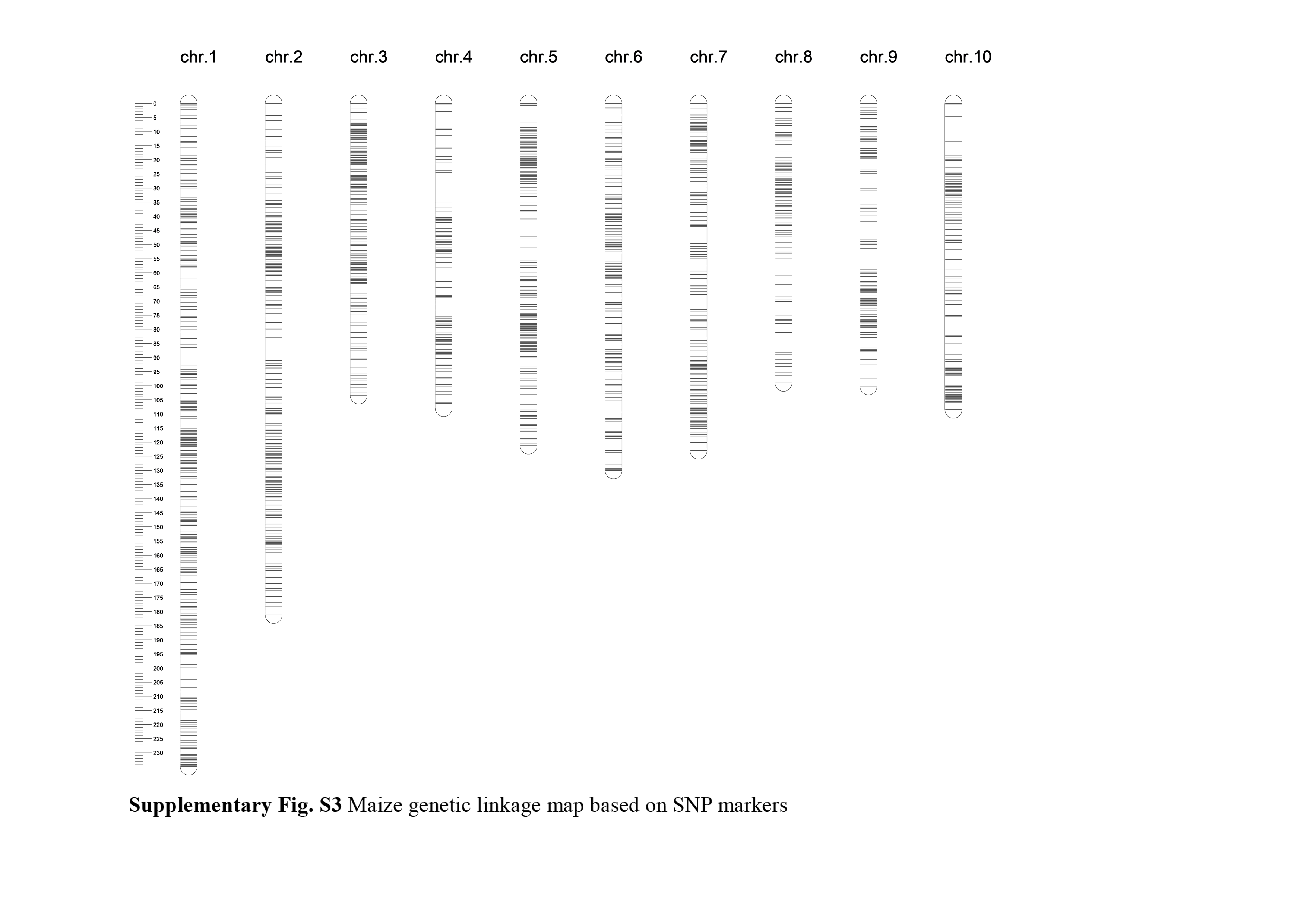

Supplement: Supplementary file 3 — Supplementary Figure S3 [file 41598_2020_69890_MOESM3_ESM.tif]

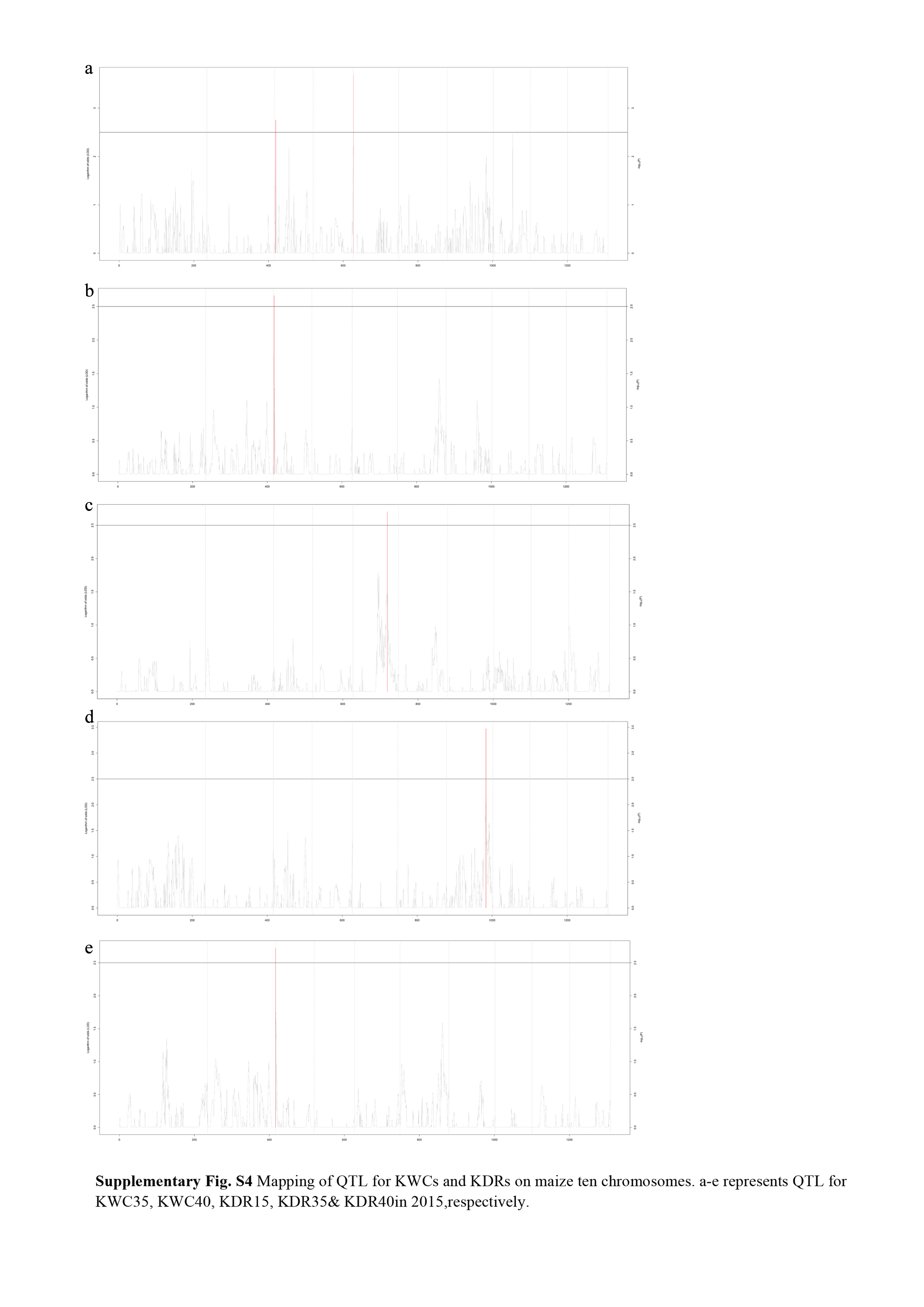

Supplement: Supplementary file 4 — Supplementary Figure S4 [file 41598_2020_69890_MOESM4_ESM.tif]

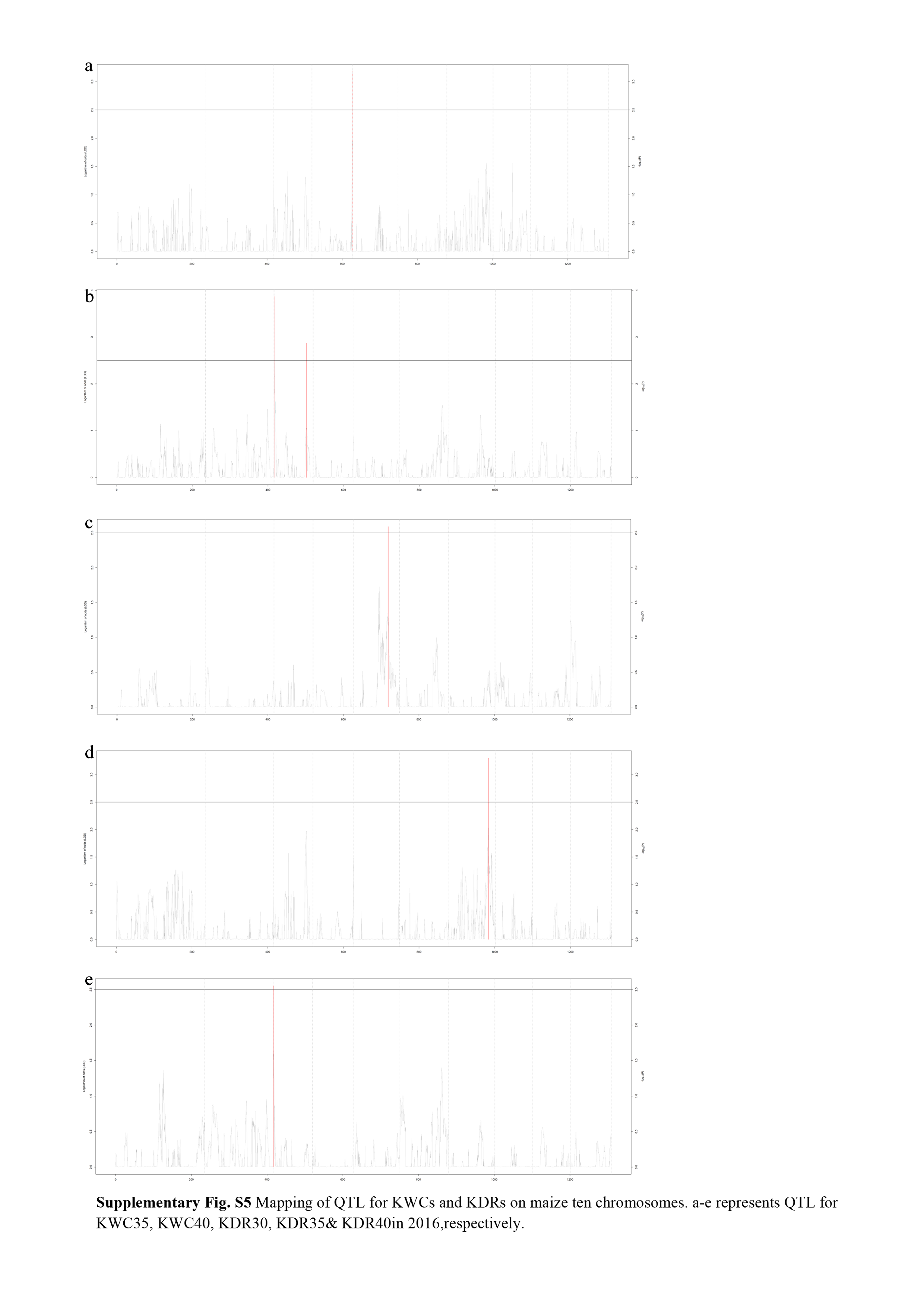

Supplement: Supplementary file 5 — SSupplementary Figure S5 [file 41598_2020_69890_MOESM5_ESM.tif]

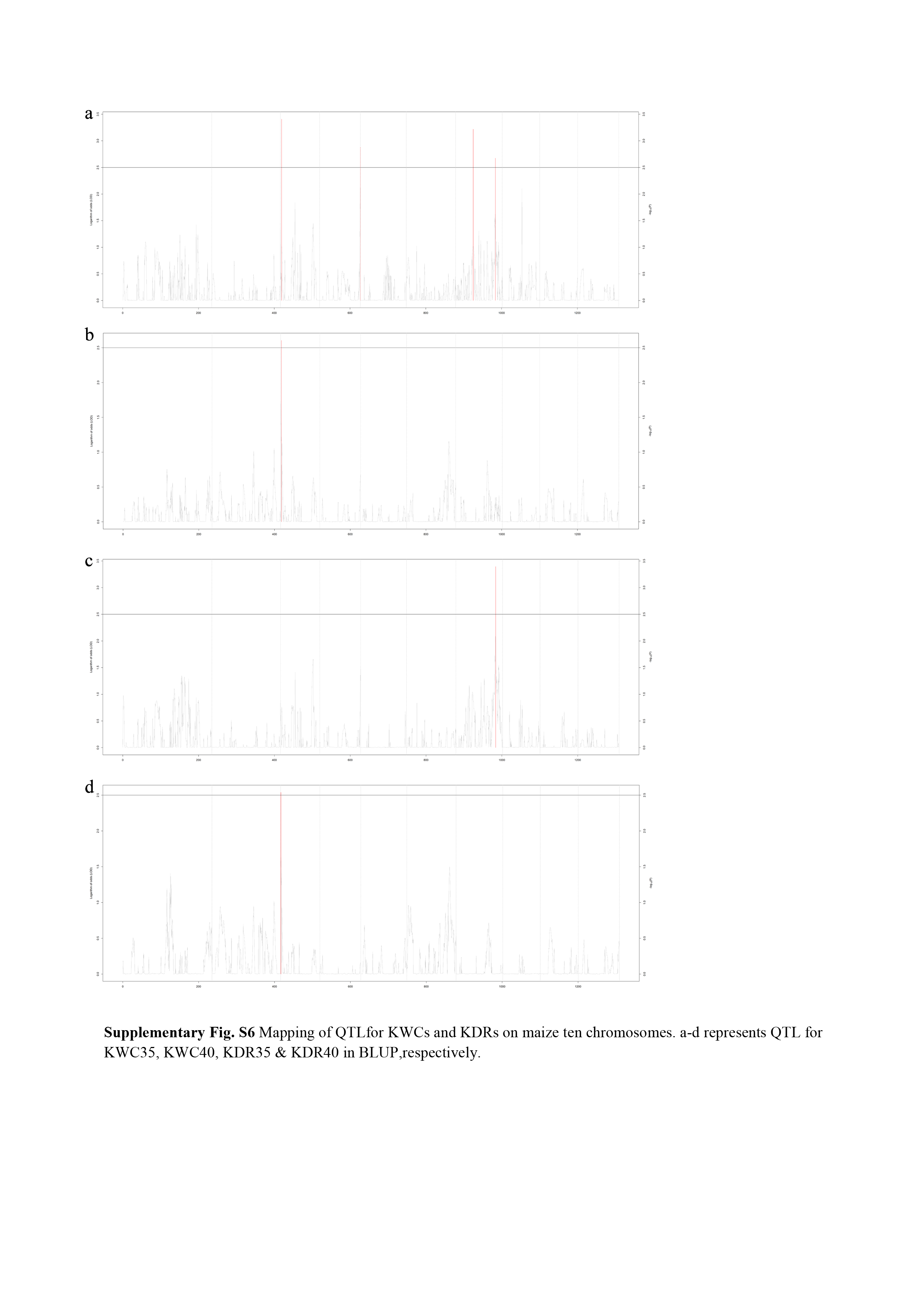

Supplement: Supplementary file 6 — Supplementary Figure S6 [file 41598_2020_69890_MOESM6_ESM.tif]

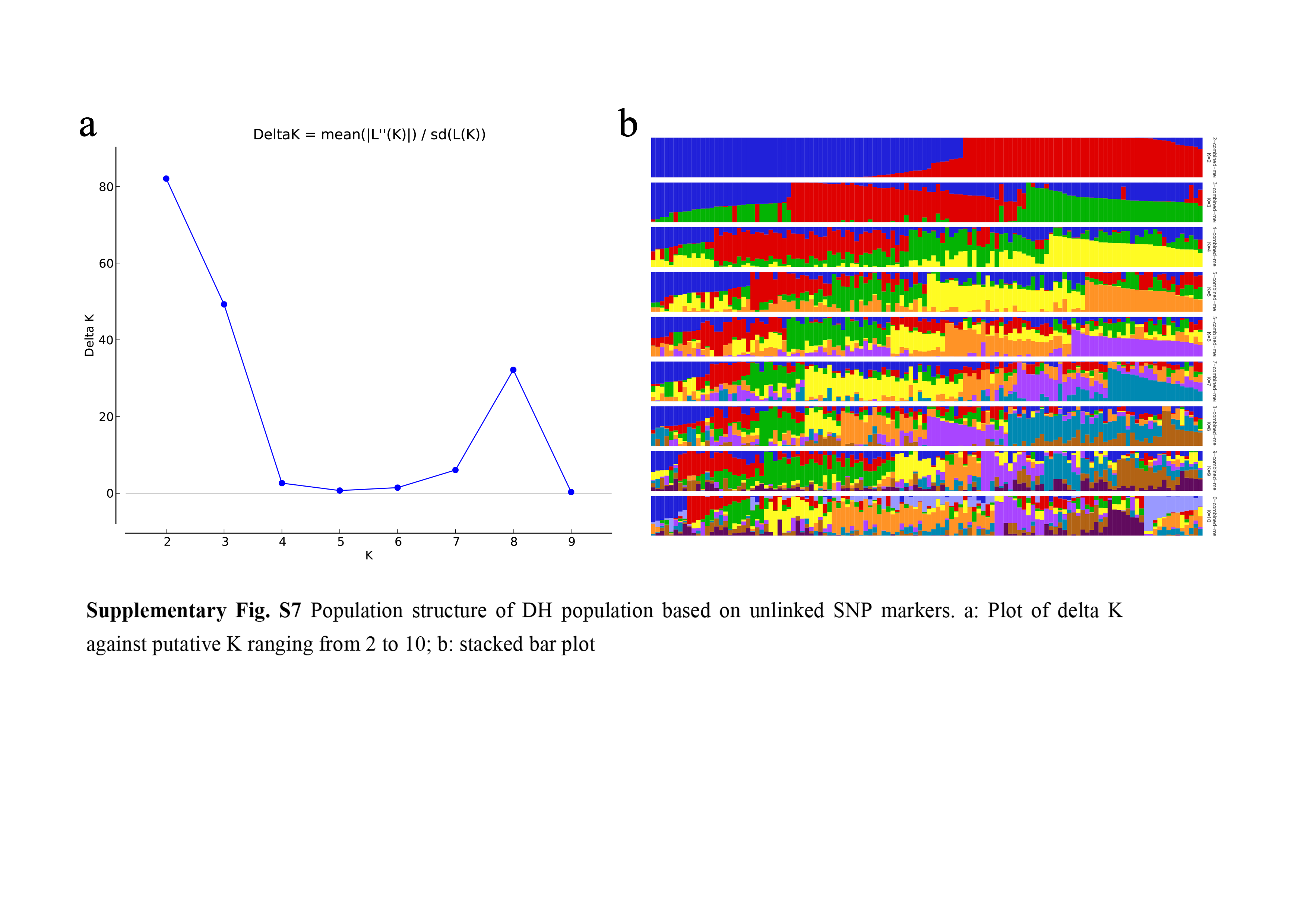

Supplement: Supplementary file 7 — Supplementary Figure S7 [file 41598_2020_69890_MOESM7_ESM.tif]

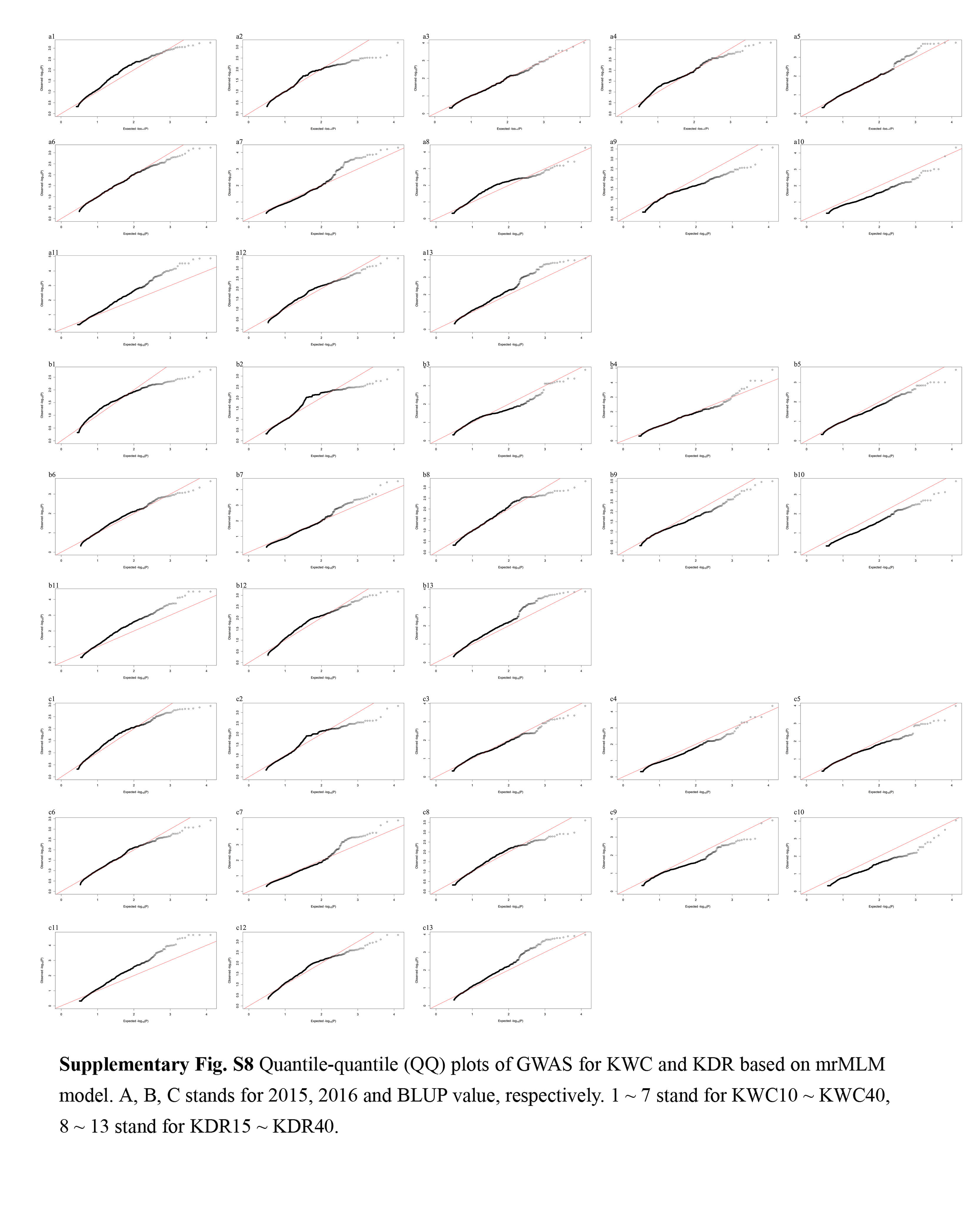

Supplement: Supplementary file 8 — Supplementary Figure S9 [file 41598_2020_69890_MOESM8_ESM.tif]
